# Circular RNA detection identifies circ*PSEN1* alterations in brain specific to autosomal dominant Alzheimer's disease

**DOI:** 10.1186/s40478-022-01328-5

**Published:** 2022-03-04

**Authors:** Hsiang-Han Chen, Abdallah Eteleeb, Ciyang Wang, Maria Victoria Fernandez, John P. Budde, Kristy Bergmann, Joanne Norton, Fengxian Wang, Curtis Ebl, John C. Morris, Richard J. Perrin, Randall J. Bateman, Eric McDade, Chengjie Xiong, Alison Goate, Martin Farlow, Jasmeer Chhatwal, Peter R. Schofield, Helena Chui, Oscar Harari, Carlos Cruchaga, Laura Ibanez, Dominantly Inherited Alzheimer Network

**Affiliations:** 1 Department of Psychiatry, Washington University in Saint Louis School of Medicine, 4444 Forest Park, Campus Box 8134, Saint Louis, MO 63110 USA; 2 NeuroGenomics and Informatics Center, Washington University in Saint Louis School of Medicine, Saint Louis, MO USA; 3 Hope Center for Neurological Disorders, Washington University in Saint Louis School of Medicine, Saint Louis, MO USA; 4 The Charles F. and Joanne Knight Alzheimer Disease Research Center, Washington University in Saint Louis School of Medicine, Saint Louis, MO USA; 5 Department of Neurology, Washington University in Saint Louis School of Medicine, Saint Louis, MO USA; 6 Department of Pathology and Immunology, Washington University in Saint Louis School of Medicine, Saint Louis, MO USA; 7 Division of Biostatistics, Washington University in Saint Louis School of Medicine, Saint Louis, MO USA; 8 Department of Neuroscience, Icahn School of Medicine at Mount Sinai, New York, NY USA; 9 Department of Neurology, Indiana University School of Medicine, Indianapolis, IN USA; 10 Department of Neurology, Massachusetts General Hospital, Boston, MA USA; 11 Neuroscience Research Australia, Sydney, Australia; 12 School of Medical Sciences, University of New South Wales, Sydney, Australia; 13 Department of Neurology, Keck School of Medicine of University of Southern California, Los Angeles, CA USA; 14 Department of Genetics, Washington University in Saint Louis School of Medicine, Saint Louis, MO USA

**Keywords:** Autosomal-dominant Alzheimer's disease, Circular RNA, Circular *PSEN1*, Differential expression, *In-silico* functional analysis, Pathway analysis, Neuroinflammation, Amyloid beta pathway

## Abstract

**Background:**

Autosomal-dominant Alzheimer's disease (ADAD) is caused by pathogenic mutations in *APP*, *PSEN1*, and *PSEN2*, which usually lead to an early age at onset (< 65). Circular RNAs are a family of non-coding RNAs highly expressed in the nervous system and especially in synapses. We aimed to investigate differences in brain gene expression of linear and circular transcripts from the three ADAD genes in controls, sporadic AD, and ADAD.

**Methods:**

We obtained and sequenced RNA from brain cortex using standard protocols. Linear counts were obtained using the TOPMed pipeline; circular counts, using python package DCC. After stringent quality control (QC), we obtained the counts for *PSEN1*, *PSEN2* and *APP* genes. Only circ*PSEN1* passed QC. We used DESeq2 to compare the counts across groups, correcting for biological and technical variables. Finally, we performed *in-silico* functional analyses using the Circular RNA interactome website and DIANA mirPath software.

**Results:**

Our results show significant differences in gene counts of circ*PSEN1* in ADAD individuals, when compared to sporadic AD and controls (ADAD = 21, AD = 253, Controls = 23—ADADvsCO: log_2_FC = 0.794, *p* = 1.63 × 10^–04^, ADADvsAD: log_2_FC = 0.602, *p* = 8.22 × 10^–04^). The high gene counts are contributed by two circ*PSEN1* species (hsa_circ_0008521 and hsa_circ_0003848). No significant differences were observed in linear *PSEN1* gene expression between cases and controls, indicating that this finding is specific to the circular forms. In addition, the high circ*PSEN1* levels do not seem to be specific to *PSEN1* mutation carriers; the counts are also elevated in APP and *PSEN2* mutation carriers. *In-silico* functional analyses suggest that circ*PSEN1* is involved in several pathways such as axon guidance (*p* = 3.39 × 10^–07^), hippo signaling pathway (*p* = 7.38 × 10^–07^), lysine degradation (p = 2.48 × 10^–05^) or Wnt signaling pathway (*p* = 5.58 × 10^–04^) among other KEGG pathways. Additionally, circ*PSEN1* counts were able to discriminate ADAD from sporadic AD and controls with an AUC above 0.70.

**Conclusions:**

Our findings show the differential expression of circ*PSEN1* is increased in ADAD. Given the biological function previously ascribed to circular RNAs and the results of our *in-silico* analyses, we hypothesize that this finding might be related to neuroinflammatory events that lead or that are caused by the accumulation of amyloid-beta.

**Supplementary Information:**

The online version contains supplementary material available at 10.1186/s40478-022-01328-5.

## Background

Alzheimer's disease (AD) is the most common cause of dementia; approximately 5.8 million Americans suffered from AD in 2019 and, by 2050, it is projected that 14 million individuals in the United States will be affected by AD [1]. AD is characterized by pathological changes in the brain: accumulation of amyloid-beta plaques (extracellular deposits of amyloid-beta peptides) and neurofibrillary tangles (NFTs, intraneuronal fibrillar aggregates of hyperphosphorylated tau). Clinically, AD is defined by gradual and progressive memory loss [2]. AD can be categorized as sporadic AD or Autosomal Dominant AD (ADAD) [3]. ADAD is caused by mutations or duplications of the amyloid precursor protein (*APP*), mutations in presenilin 1 (*PSEN1*), or mutations in presenilin 2 (*PSEN2*), an autosomal dominant inheritance within family members for more than two generations, and an onset earlier than 65 years old [4, 5]. More than 400 mutations have been reported on these three genes, but *PSEN1* harbors the most mutations, which are also associated with the youngest age at onset with affected individuals typically being 30–50 years old [4, 6–8]. In fact, *PSEN1* mutations have also been reported in late-onset AD [9]. Even though ADAD is rare (< 0.5% of all AD cases) [10], these cases have provided unique insights into the pathobiology of the disease, especially the formulation of the amyloid hypothesis: that accumulation of amyloid-beta aggregates initiates the pathologic process of AD. All three ADAD-causing genes are part of the amyloid-beta processing pathway. However, neuropathological studies have shown that there are common and distinct pathological characteristics [11]. Both ADAD and AD present neuronal loss, neurofibrillary tangles, amyloid plaques and cerebral amyloid angiopathy among others, but ADAD show, for example, cottonwool plaques, more severe cerebral amyloid angiopathy, more common intracerebral hemorrhage or higher abundance of Lewy bodies [5, 12].

Recently, studies screening the whole genome, or the brain transcriptome have been instrumental in elucidating downstream genes and pathways implicated in disease, highlighting the importance of studying AD beyond the amyloid pathway [13–18]. However, most of these studies have been focused on sporadic AD, so their findings cannot always be extrapolated to Mendelian forms of AD; differences between these two forms of the disease are well known [5, 12, 19]. Several studies focused on ADAD have been limited to genetic studies focused on families [6, 20, 21] and animal studies aiming to understand the amyloid cascade hypothesis [22]. Studies involving large diverse ADAD cohorts are limited.

Circular RNAs are a family of non-coding RNAs that result from backsplicing events (the 3’ end of the transcript links covalently to the 5’ forming a loop) [23, 24]. The knowledge of circular RNAs is still limited, but it is thought that they are implicated in the regulation of microRNAs via sequestration, leading to a loss of function of the microRNA [20, 23–25]. Circular RNAs are highly expressed in the nervous system and especially in synapses [20]. Dysregulation of circular RNAs has already been shown for several central nervous diseases, including AD, Parkinson’s disease, and traumatic brain injury [20, 25–27]. CircRNA were systematically screened in brain samples from AD compared to controls [20]. They successfully identified more than 100 circRNAs associated with AD status and disease severity measured by Braak neurofibrillary tangles (NFT) and Clinical Dementia Rating (CDR®) [28]. When subsetting the analyses to ADAD, 236 circRNAs were found to be dysregulated; 56 of them independently of the severity measured by Braak NFT The circRNAs associated with both ADAD and AD showed larger effect sizes in ADAD than in AD. However, no specific analyses regarding the circular forms of the ADAD genes were performed. In this study, we used bulk RNA-seq to postmortem parietal cortex samples from controls, sporadic AD, and ADAD, we investigated the gene expression profiles of linear and circular transcripts of the ADAD genes to determine their possible involvement in the pathobiology of ADAD.

## Methods

### Study population

The discovery phase included bulk RNA-seq data from parietal cortex samples from non-Hispanic white (NHW) participants: 17 ADAD participants from the Dominantly Inherited Alzheimer Network (DIAN) (14 *PSEN1* and three *APP* mutation carriers), 59 sporadic AD participants and ten control participants from the Knight-ADRC at Washington University in Saint Louis. The replication phase included four ADAD cases from DIAN (two *PSEN1*, one *PSEN2*, and one *APP mutation* carriers), and 194 sporadic AD cases and 13 controls from the Knight-ADRC. Demographic information can be found in Table 1, and neuropathological characteristics of the included brains is specified in Additional file 1: Table S1. Finally, we leveraged the Mount Sinai Brain Bank (MSBB) dataset (syn3157743) for replication of the sporadic AD findings (Additional file 1: Table S2). MSBB contains brain RNA-seq data from different brain regions. From Brodmann area (BM) 10 (frontal pole): 143 AD, 29 Controls; from BM22 (superior temporal gyrus): 134 AD, 26 Controls; from BM36 (parahippocampal gyrus): 123 AD, 24 Controls; from (BM) 44 (inferior frontal gyrus): 132 AD, 25 Controls.

**Table 1:** Demographic characteristics of the discovery and replication datasets

	Discovery			Replication			Joint dataset		
	ADAD	AD	Controls	ADAD	AD	Controls	ADAD	AD	Controls
Sample size	17	59	10	4	194	13	21	253	23
Age at death (years)	52.0 (38.8–88.2)	84.0 (72.0–96.1)	92.5 (75.7–103.4)	58.0 (42.1–75.6)	83.0 (68.0–98.0)	87.0 (80.0–101.0)	54.0 (39.0–88.0)	83.0 (68.0–98.0)	90.0 (79.1–104.0)
Sex (% males)	71%	49%	30%	75%	39%	38%	71%	41%	35%
Age at onset (years)	39.0 (28.6–76.0)	75.0 (58.8–90.2)	–	62.0 (52.1–71.9)	73.0 (55–92.0)	–	51.0 (28.9–75.8)	73.0 (55.5–92.0)	–
*PSEN1* mutation (% of carriers)	82%	0%	0%	50%	0%	0%	76%	0%	0%
PMI (hours)	9.0 (4.2–30.8)	11.6 (4.4–22.5)	10.5 (5.1–19.4)	10.3 (4.7–14.9)	12.1 (4.0–23.0)	9.1 (3.8–16.2)	9.0 (4.5–26.5)	12.0 (4.0–22.7)	9.5 (4.5–20.5)
CDR® at death	2.0 (1.5–3.0)	3.0 (3.0–3.0)	0.3 (0–0.5)	3.0 (2.5–3.0)	3.0 (2.0–3.0)	0 (0–0)	2.5 (2.0–3.0)	3.0 (2.0–3.0)	0 (0–0.25)

*ADAD* autosomal dominant Alzheimer's disease, *AD* sporadic Alzheimer's disease, *PSEN1* presenilin1, *PMI* post-mortem interval, *CDR*® clinical dementia rating. Age at Death, Age at Onset, and PMI are expressed as the median value with 95% Inter Quartile Interval; CDR® at Death is expressed as the median value with 75% Inter Quartile Interval

### Library preparation and sequencing

The data generation for the discovery dataset has been previously published [20, 21]. We followed the same protocol to generate the replication dataset. Briefly, total RNA was obtained from frozen parietal cortex tissue using the Tissue Lyser LT and RNeasy Mini Kit (Qiagen). After quality control, libraries were generated using TruSeq Stranded Total RNA Sample Prep with Ribo-Zero Gold kit (Illumina). Eighty million 2 × 150 bp reads were generated on average for each sample using an Illumina HiSeq 4000. For the replication dataset, we obtained the RNA from frozen brain tissue with the Maxwell RSC simplyRNA tissue kit (Promega). After quality control, TruSeq Stranded Total RNA Sample Prep with Ribo-Zero Gold kit (Illumina) was used to generate the libraries. Thirty-five million 2 × 150 bp reads were generated on average for each sample using an Illumina NovaSeq 6000.

### RNA-seq quality control, alignment, and circular RNAs detection

Both datasets were processed and aligned separately following similar pipelines to the ones previously published by our group [20, 21]. Genome reference and gene models were selected following the TOPMed pipeline (https://github.com/broadinstitute/gtex-pipeline/blob/master/TOPMed_RNAseq_pipeline.md). Reference genome GRCh38 and GENCODE 33 annotation, including the addition of ERCC spike-in annotations were used. We excluded ALT, HLA, and Decoy contigs from the reference genome due to the lack of RNA-seq tools that allow proper handling of these regions. To obtain the linear counts we followed standard guidelines. Briefly, the raw reads were aligned to the human reference genome (GRCh38) using STAR (v.2.7.1a) [29]. We evaluated the quality of the alignment using sequencing metrics such as reads distribution, ribosomal content or alignment quality provided by STAR [29] using Picard tools (v.2.8.2) [30]. Gene expression was quantified using Salmon (v.1.2.0) [31] and the GENCODE reference genome (GRCh38). All transcripts or genes with less than ten reads in more than 90% of the individuals were removed.

To obtain the circular RNA counts, all raw reads were first aligned to the human reference genome (GRCh38) using STAR [29] in chimeric alignment mode. The remaining alignment parameters were selected specifically for circular RNA detection as suggested by the developers of the circular RNA calling software DCC [32]. Circular RNA detection, annotation and quantification was performed using DCC (v.0.4.8). We excluded any circRNA that had missing counts in more than 25% of the samples. As part of the general quality control, circRNAs that were not present in at least three samples, with a minimum of three counts in at least one of them, were removed. Additionally, we removed any circRNA with missing counts in more than 75% of the samples. Then we proceeded to extract the linear and circular forms corresponding to the three ADAD genes (*APP, PSEN1* and *PSEN2*) from each dataset. For all three datasets, only circ*PSEN1* could be detected; circ*APP* and circ*PSEN2* were not detected. Consequently, the analyses were focused on linear *PSEN1* and circ*PSEN1.* The species of circ*PSEN1* varied depending on the dataset and the brain region for the MSBB. Overall, seven circ*PSEN1* species were detected by DCC; but four of them (hsa_circ_0008521, hsa_circ_0003848, hsa_circ_0007013, hsa_circ_0002564) were commonly detected in all three datasets and most of the brain areas (except hsa_circ_0008521 for BM10, hsa_circ_0007013 for BM22 and BM44).

### Statistical analyses

We tested if the levels of circular *PSEN1* (circ*PSEN1*) and linear *PSEN1* were different among groups by comparing AD and control participants, ADAD and AD participants, and ADAD and control participants in both the discovery and the replication datasets. The same analysis of circ*PSEN1* was also performed between sporadic AD participants and control subjects in the MSBB dataset (no ADAD participants were available in the MSBB dataset). We also investigated which specific *circPSEN1* transcripts were predominant in each group and dataset. After normalization of the counts, differential expression (DE) analyses were performed specifically for circ*PSEN1* and linear *PSEN1* using DEseq2 version 1.22.2 [33] to determine significance. Any association with *p* value < 0.05 was considered significant. All DE analyses were adjusted for postmortem interval (PMI), RNA quality as measured by median transcript integrity number (TIN) [34] and sex. We also tested if circ*PSEN1* was associated to Braak NFT or age at death to investigate if our findings were driven by disease severity as previously described [20]. Briefly, the variable of interest was added to the model to evaluate the effect on the p-value, effect size and direction of the circ*PSEN1* association.

Due to the moderate size of our sample, we combined the discovery and replication datasets, since they were processed using the same pipeline, and performed a joint analysis adding dataset to the model to adjust for possible differences in the DE analysis.

### *In-silico* functional study

To investigate the biological function of circ*PSEN1*, we accessed the Circular RNA Interactome website [35] to predict which miRNAs have the potential to target any of the circ*PSEN1* species identified in our datasets. Then, we used the DIANA mirPath software version 3 [36] to identify which genes and pathways were regulated by the identified miRNA using the microT-CDS algorithm and the Kyoto Encyclopedia of Genes and Genomes (KEGG). Finally, we investigated if any of the common genes listed in the pathways identified by the DIANA mirPath software version 3 were differentially expressed in the ADAD cases compared to controls or sporadic AD cases in the discovery dataset.

### Discriminative ability of circ*PSEN1*

To evaluate if circ*PSEN1* can discriminate ADAD from the other brains, we used three binomial regression models built using three different circ*PSEN1* normalized counts: aggregate counts, hsa_circ_0008521, and hsa_circ_0003848 counts to classify ADAD vs. controls, ADAD vs. AD, and AD vs. controls in both the discovery and the replication datasets separately. We used the discovery dataset to train the models, and the replication dataset to validate them. We then evaluated the model performance through receiver operating characteristic curves (ROCs) and areas under the ROC curve (AUCs). The binomial regression models and the receiver operating characteristic curve analyses were performed using R packages stats version 3.5.2 and ROCR version 1.0-7.

## Results

### circ*PSEN1* is more abundant in ADAD than sporadic AD and controls

**Fig. 1:**
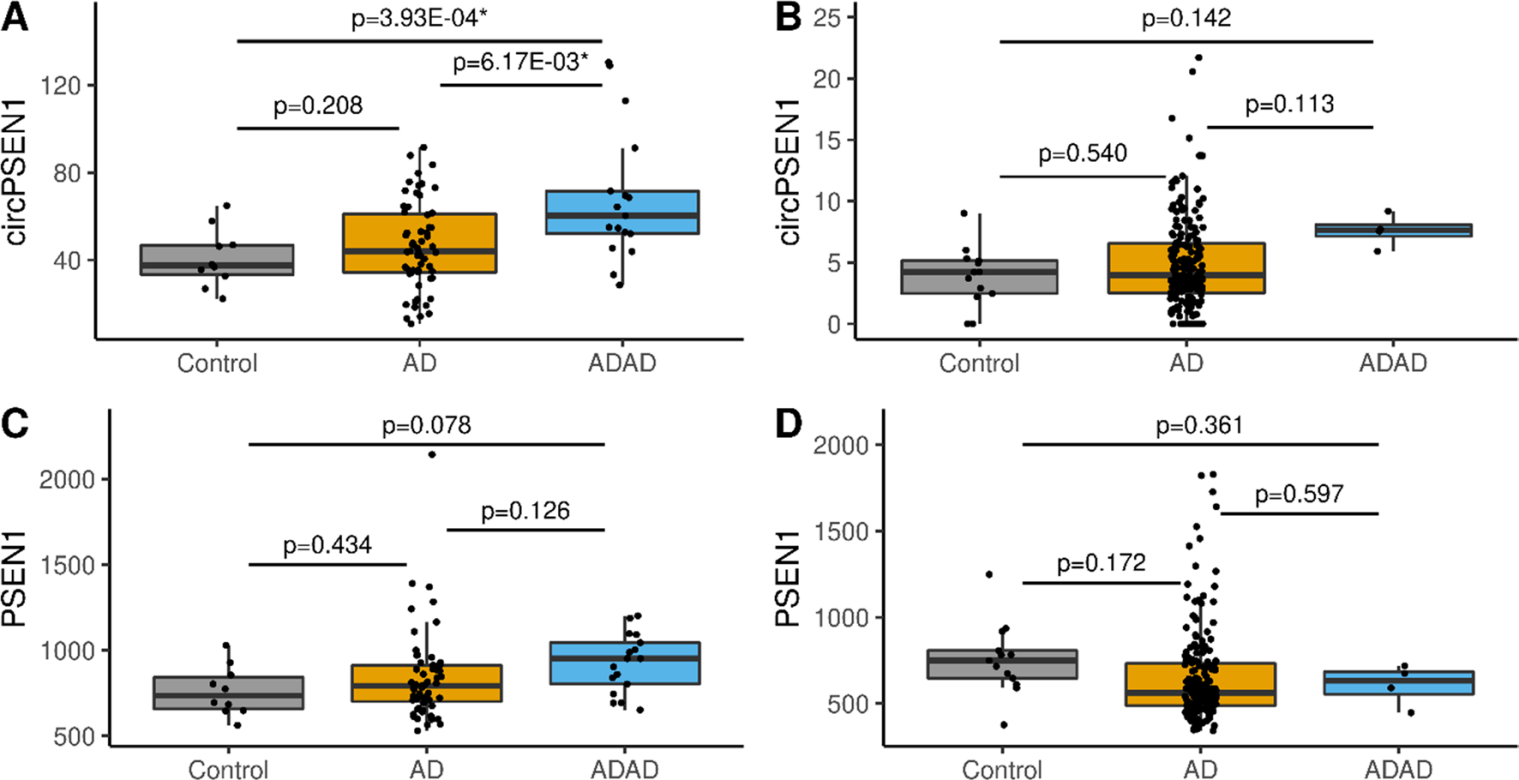
Comparison of the circular *PSEN1* normalized counts in the discovery (**A**) and replication (**B**) datasets and the normalized counts for the linear forms of *PSEN1* in the discovery (**C**) and replication (**D**) datasets for Controls (grey), AD (Alzheimer's disease—ocher) and ADAD (autosomal dominant Alzheimer's disease—blue)

The circ*PSEN1* normalized counts were significantly higher in ADAD cases (N = 17) compared to controls (N = 10; *p* = 3.93 × 10^–04^, log_2_FC = 0.80) and sporadic AD cases (N = 59; *p* = 6.17 × 10^–03^, log_2_FC = 0.52), but did not differ between AD cases and controls (*p* = 0.21, log_2_FC = 0.29) (Table 2 and Fig. 1A) in the discovery dataset. The trend was also observed in the replication dataset in all three comparisons (log_2_FC_ADADvsCO_ = 0.72; log_2_FC_ADADvsAD_ = 0.74; log_2_FC_ADvsCO_ = 0.17) (Table 2 and Fig. 1B). Due to the limited sample size of the ADAD group, we do not have enough statistical power to detect differences in the replication dataset (24 ADAD samples are required to have 80% power with a probability of type I error of 0.05). Consequently, we performed a joint analysis of the two datasets. A more significant association was found in the join analyses than in the discovery dataset, indicating that the higher expression level of circ*PSEN1* specific to ADAD than sporadic AD cases and controls (*p*_ADADvsCO_ = 1.63 × 10^–04^, log_2_FC = 0.79; *p*_ADADvsAD_ = 8.22 × 10^–04^, log_2_FC = 0.60; *p*_ADvsCO_ = 0.30, log_2_FC = 0.19; Table 2).

**Table 2:** Differential expression results

	Circular *PSEN1*		Linear *PSEN1*	
	log_2_FC	*P* value	log_2_FC	*P* value
*ADAD versus controls*				
Discovery	0.799	**3.93 × 10**^**–04**^	0.217	0.078
Replication	0.722	0.142	− 0.235	0.361
Joint Analysis	0.794	**1.63 × 10**^**–04**^	0.261	0.062
*ADAD versus AD*				
Discovery	0.522	**6.17 × 10**^**–03**^	0.163	0.126
Replication	0.735	0.113	− 0.137	0.597
Joint Analysis	0.602	**8.22 × 10**^**–04**^	0.128	0.332
*AD versus controls*				
Discovery	0.289	0.208	0.101	0.434
Replication	0.171	0.540	− 0.199	0.172
Joint Analysis	0.186	0.303	− 0.082	0.442

Bold values denote nominal significance (*P* < 0.05)

*ADAD* autosomal dominant Alzheimer's disease; *AD* sporadic Alzheimer's disease; *PSEN1 *presenilin 1

We leveraged the MSBB dataset to confirm that there were no differences between the normalized counts of circ*PSEN1* between AD cases and controls. circ*PSEN1* is not differentially expressed in any of the four brain regions (BM10, BM22, BM36, BM44) available in the MSBB dataset (Additional file 2: Fig. S1 and Additional file 1: Table S3), which is consistent with our finding that higher circ*PSEN1* expression is specific to ADAD cases.

Similar to RNA-seq, circ*PSEN1* total count is the result of the addition of all the counts from the different species of circ*PSEN1*. We investigated if the differences observed between ADAD and AD or controls were the result of an overall increase or the increase of a specific species. Seven circ*PSEN1* species were found in the discovery and replication dataset (Table 3). All of them are exon derived except one that is intron–exon derived (circ*PSEN1*- 73147795–73165413). Considering the four species commonly detected in both datasets, the most abundant are hsa_circ_0008521, hsa_circ_0003848, and hsa_circ_0002564; all three abundant species are significantly different between ADAD and CO (*p* = 2.57 × 10^–04^, 1.65 × 10^–03^, 0.036, log_2_FC = 0.96, 0.87, 0.70) and ADAD and AD (*p* = 2.20 × 10^–03^, 6.97 × 10^–03^, 8.00 × 10^–03^, log_2_FC = 0.72, 0.62, 0.64). No significant differences were found between disease status for hsa_circ_0007013 (Table 3 and Additional file 3: Fig. S2), suggesting that all three circRNA species are driving the association. No significant differences were found between AD and controls for the two species detected in the MSBB dataset in any brain region (Additional file 1: Table S3). We also investigated the relationship among these three species using correlation tests. We observed high correlation between hsa_circ_0008521 and hsa_circ_0003848 in the discovery (r^2^ = 0.80, *p* < 2.20 × 10^–16^), replication (r^2^ = 0.31, *p* = 4.22 × 10^–06^), and MSBB (BM22—r^2^ = 0.42, *p* = 3.44 × 10^–08^|BM36—r^2^ = 0.58, *p* = 7.79 × 10^–15^|BM44—r^2^ = 0.71, *p* = 2.2 × 10^–16^) datasets. Hsa_circ_0002564 shows weak correlations with the other two circ*PSEN1* species even though some of them are nominally significant (with hsa_circ_0008521 only in the discovery dataset—r^2^ = 0.25, *p* = 0.02, and with hsa_circ_0003848 in the discovery—r^2^ = 0.26, *p* = 0.01 and in the BM22 of the MSBB dataset—r^2^ = 0.31, *p* = 8.15 × 10^–05^). This results suggest that hsa_circ_0008521 and hsa_circ_0003848 seem to share the same mechanism of dysregulation, which might be different from hsa_circ_0002564.

**Table 3:** Species of *circPSEN1* characterization and differences across groups

circ*PSEN1* species		Discovery			Replication			Joint		
		ADADvsCO	ADADvsAD	ADvsCO	ADADvsCO	ADADvsAD	ADvsCO	ADADvsCO	ADADvsAD	ADvsCO
hsa_circ_0008521	*P* value	**1.760 × 10**^**–03**^	**6.691 × 10**^**–03**^	0.278	0.500	0.368	0.453	**2.572 × 10**^**–04**^	**2.196 × 10**^**–03**^	0.244
	log_2_ FC	0.923	0.594	0.300	0.861	0.794	0.420	0.955	0.719	0.311
hsa_circ_0003848	*P* value	**0.017**	**0.025**	0.180	0.171	0.190	0.876	**1.647 × 10**^**–03**^	**6.965 × 10**^**–03**^	0.519
	log_2_ FC	0.756	0.455	0.295	1.731	1.325	− 0.100	0.871	0.618	0.164
hsa_circ_0007013	*P* value	0.819	0.854	0.386	0.847	0.725	0.875	0.859	0.638	0.566
	log_2_ FC	− 0.298	− 0.124	0.766	0.628	0.665	0.172	0.195	0.319	0.381
circ*PSEN1 *73147795–73165413	*P* value	–	–	–	0.833	0.992	0.929	–	–	–
	log_2_ FC	–	–	–	− 0.819	− 0.104	− 0.540	–	–	–
hsa_circ_0002564	P value	0.065	**4.848 × 10**^**–04**^	0.706	0.580	0.555	0.541	**0.036**	**7.998 × 10**^**–03**^	0.388
	log_2_ FC	0.740	0.858	0.126	0.412	0.344	0.208	0.701	0.636	0.211
hsa_circ_0008218	P value	–	–	–	0.943	0.667	0.801	–	–	–
	log_2_ FC	–	–	–	− 0.277	0.733	− 0.255	–	–	–
hsa_circ_0032509	P value	0.816	0.840	–	–	–	–	–	–	–
	log_2_ FC	0.717	0.640	0.207	–	–	–			

Bold values denote nominal significance (*P* < 0.05)

*ADAD* autosomal dominant Alzheimer's disease, *AD* sporadic Alzheimer's disease, *CO* control, *PSEN1 *Presenilin 1

To ensure that the association was not driven by disease severity or age at death, we tested if Braak NFT score or age at death were influencing the association of circ*PSEN1* with ADAD. We observed no significant changes in the results (Additional file 1: Table S4), suggesting that these findings are not due to pathology severity or the age of the individual.

### Circular *PSEN1* is independent from linear *PSEN1*

We then investigated if the association between ADAD and circ*PSEN1* was also observed in the linear form of *PSEN1.* No significant differences were found (Table 2, Fig. 1C, D).

We then tested the independence of the normalized counts for the linear and circular forms of *PSEN1.* Significant correlation was observed for the ADAD individuals or the controls (Additional file 4: Fig. S3). The correlation between the linear *PSEN1* and the circ*PSEN1* was weak (R_Discovery_ = 0.42; R_Replication_ = 0.20), even though it was nominally significant (*p*_Discovery_ = 8.20 × 10^–04^, *p*_Replication_ = 3.90 × 10^–03^) in the AD group. To assess if this correlation was affecting our results in the AD group, linear *PSEN1* was added to the differential expression analysis as previously described [37]. The changes of circ*PSEN1* were still significant (*p* = 1.71 × 10^–04^), even when adjusting for linear *PSEN1*. This result suggests that even though the correlation of linear and circular *PSEN1* was nominally significant, the linear and circular forms are independent. Similar results were observed for correlation analyses using for *PSEN1* species against linear counts (Additional file 5: Fig. S4).

### ADAD individuals have higher circ*PSEN1* counts independently of the mutation

**Fig. 2:**
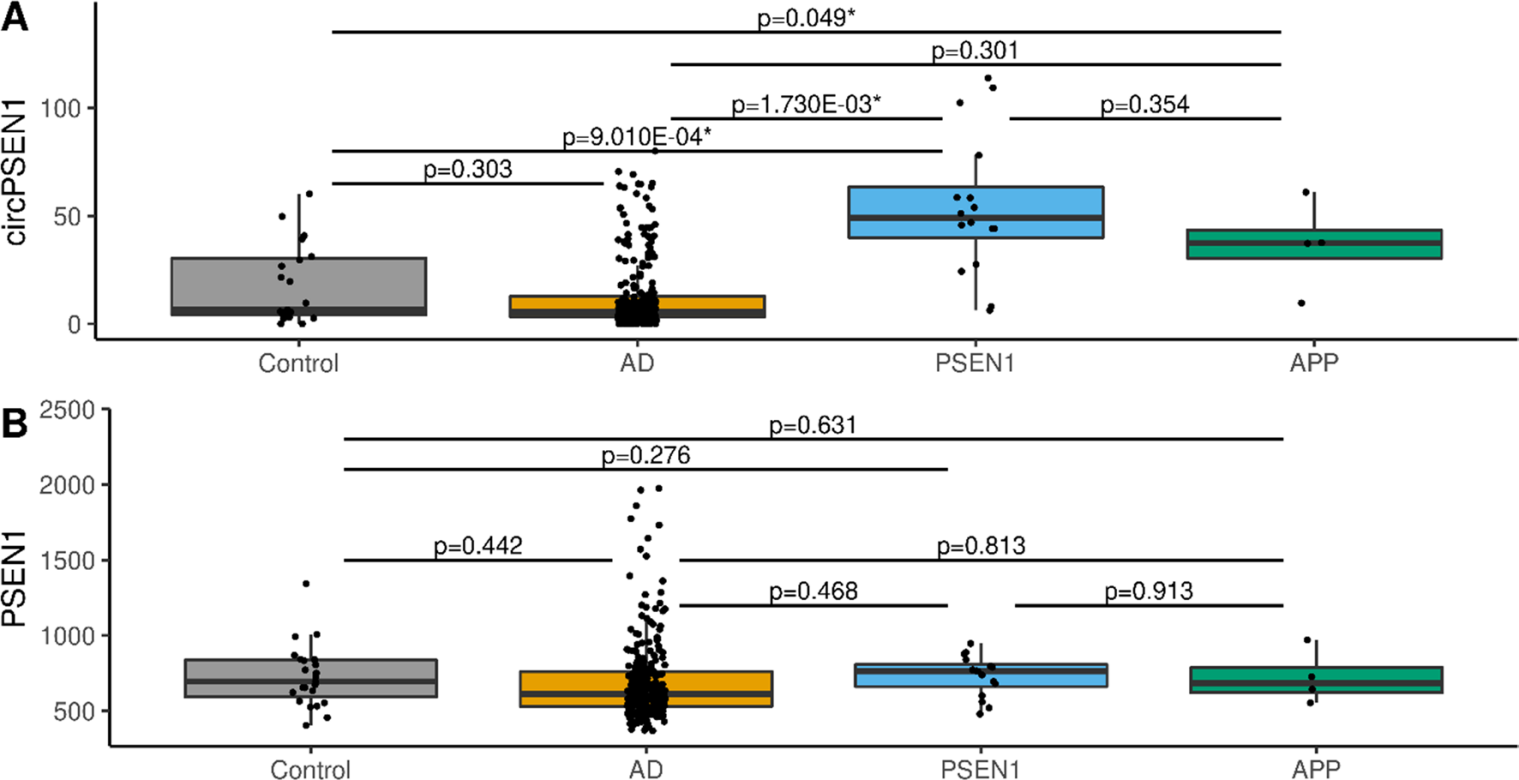
Comparison of the normalized counts of circular *PSEN1* (**A**)/linear *PSEN1* (**B**) between controls, AD, and different mutation carriers in the joint dataset—Controls (grey), ADs (Alzheimer's disease—ocher), *PSEN1* mutation carriers (blue), and *APP* mutation carriers (green)

ADAD mutations are most prevalent in *PSEN1* among individuals in both the discovery and the replication datasets. Thus, we evaluated if the high expression levels of circ*PSEN1* were unique to *PSEN1* mutation carriers. In the joint dataset (Fig. 2A), the normalized counts of circ*PSEN1* between *PSEN1* mutation carriers (N = 16) and *APP* mutation carriers (N = 4) are not significantly different (log_2_FC = 0.332, *p* = 0.354). When compared to AD cases (N = 253) or controls (N = 23), *PSEN1* mutation carriers showed increased levels of circ*PSEN1* (log_2_FC = 0.634, *p* = 1.730 × 10^–03^, and log_2_FC = 0.787, *p* = 9.010 × 10^–04^). *APP* mutation carriers also showed higher counts of circ*PSEN1* when compared to controls (log_2_FC = 0.597, *p* = 0.049). Due to the limited sample size of *PSEN2* mutation carriers (N = 1), no statistical test was performed. No differences were found among mutations carriers regarding linear *PSEN1* normalized counts (Fig. 2B).

### *In-silico* analyses functional annotation

We found the three most abundant species of circ*PSEN1* in our samples to be hsa_circ_0008521, hsa_circ_0003848, and hsa_circ_0002564, with the first two most likely driving the overall signal. CircRNAs have been reported to regulate gene expression by sequestering miRNA. Because the biological function of these circ*PSEN1* species has not been described, we explored if the biological function of circ*PSEN1* might be elucidated by those miRNAs targeted to them. Using the Targetscan prediction tool [38] from the CircInteractome database [35], we identified 26 miRNAs that could potentially target to the three most abundant species of circ*PSEN1*. We used them as input in the microT-CDS tool of the DIANA mirPath v.3 software to elucidate which pathways are potentially regulated by the identified miRNA. Several pathways were found significantly associated with these 26 miRNAs: axon guidance (*p* = 3.39 × 10^–07^); hippo signaling pathway (*p* = 7.38 × 10^–07^); lysine degradation (*p* = 2.48 × 10^–05^); and Wnt signaling pathway (*p* = 5.58 × 10^–04^) among other KEGG pathways (Additional file 1: Table S5). We identified 31 genes that were common in the top ten KEGG pathways (Additional file 1: Table S6); the counts of two of them were found significantly lower in ADAD brains compared to AD brains. *FZD4* and *RAF1* were nominally significant in the discovery (*p* = 0.045; *p* = 1.07 × 10^–03^) and replication (*p* = 1.55 × 10^–03^; *p* = 0.049) datasets. Both genes are related to the transmission of chemical signals between the cell surface and the nucleus.

### Circular PSEN1 normalized counts can discriminate ADAD

The predictive ability of *circPSEN1* counts (aggregate, hsa_circ_0008521, and hsa_circ_0003848) was evaluated using ROCs and AUCs (Additional file 6: Fig. S5). Aggregated and individual normalized counts of circ*PSEN1* show no predictive ability for AD vs. CO. However, *circPSEN1* showed good predictive power for ADAD vs. AD. The AUC for the aggregated counts in the discovery dataset was 0.71; that in the replication dataset was 0.81. When we evaluated the predictive power of the two circ*PSEN1* species separately, hsa_circ_0008521 seemed to have slightly better predictive power than hsa_circ_0003848. Yet, the aggregate counts of circ*PSEN1* seemed to show a more robust discriminative power, which generated similar AUCs across datasets.

The discriminative power of circ*PSEN1* increased when we attempted to classify ADAD vs. CO, with an AUC of 0.81 in the discovery dataset and an AUC of 0.85 in the replication dataset for the aggregated counts of circ*PSEN1*. The trends were very consistent for both datasets for all three predictions despite the differences in sample size. This result suggests that, even though the most abundant circ*PSEN1* species seem to have more discriminative power, the less abundant ones (hsa_circ_0007013, hsa_circ_0002564, hsa_circ_0008218, hsa_circ_0032509) are also contributing since the discriminative power of the aggregate counts is more stable in the two datasets.

## Discussion

In this study, we provide evidence that the transcriptional signatures differ between ADAD and AD brains. By analyzing the largest dataset to date of ADAD brains, we have found that circ*PSEN1* expression is increased in ADAD brains but not in AD cases or controls, a result which is independent of age and disease severity (as measured by Braak NFT score). Our results show that this increased expression is not specific to *PSEN1* mutation carriers, as similar results were observed in *APP* mutation carriers, or due to linear *PSEN1,* suggesting a biological mechanism specific to the *circPSEN1*.

A previous study [20], demonstrated that expression changes of circRNAs in pre-symptomatic AD, sporadic AD, and ADAD are different and not always related to severity of the disease. They found more than 100 circRNAs dysregulated in the context of AD, demonstrating the involvement of circRNA in the pathobiology of AD. In fact, they found even more circRNA dysregulated in their comparison of ADAD participants versus controls. In the present study we add evidence to the importance of the dysregulation of circRNAs. Dube et al., identified that circRNAs that were dysregulated in both AD and ADAD, presented with larger effect sizes in ADAD. In here, we found that circ*PSEN1* that is related to the amyloid pathway is uniquely dysregulated in ADAD participants. This emphasizes the importance of studying not only the molecular similarities between AD and ADAD, but also the differences.

It has been demonstrated that circular RNAs are generated through the spliceosome, suggesting that additional to the miRNA regulation through their sponge function, circular RNA generation is one of the mechanisms that regulates the production of linear RNA [39]. On top of that, spliceosomal proteins have been reported to aggregate with tau tangles [40] and to be down-regulated in the presence of amyloid-beta_42_ [41]. The production of circ*PSEN1* could be due to the mutations present in *PSEN1* via spliceosome alterations. Among the *PSEN1* mutations in this study, p.H163R is located at the boundaries of hsa_circ_0002564. This might explain the dysregulation of this particular circular RNA. However, this mutation was present in only four of the ADAD brain samples, suggesting that other regulatory events are taking place in the ADAD individuals unrelated to this mutation. In fact, previous studies have demonstrated that introns regulate the biogenesis of circular RNA. Given that most of the *PSEN1* mutations are located within the exons, it is likely that this might not be the biological explanation.

Our *in*-*silico* functional analysis predicted 26 miRNAs that could bind the three abundant circ*PSEN1* species. These 26 miRNAs are significantly associated with several pathways, including wnt, hippo and axon guidance pathways that have been previously related to the development of AD and to neuroinflammation [42–45]. Among the 26 identified miRNAs, miR-144 has previously been associated with AD [46]; in fact, miR-144-3p targets *APP,* significantly inhibiting protein expression [47]. The overexpression of miR-433 targets *JAK2* (janus kinase 2) which contributes to the progression of AD by inhibition of amyloid-beta-induced neuronal viability [48]. Additionally, miR-566 [49] and miR-885-5p [50] were also found to be dysregulated in AD. Finally, miR-655 inhibited the inflammatory response of microglia by targeting *TREM2* (triggering receptor expressed on myeloid 2) [51], which is known to affect amyloid and tau pathologies. Our finding adds evidence to the fact that AD is not restricted to neurons but involves several mechanisms including inflammation [52]. Given the involvement of microglia in the inflammatory process, axon guidance [53], and the role of wnt pathway [45], the presence of circ*PSEN1* might be originating from the microglia. Analyses of circ*PSEN1* using publicly available RNAseq data from IPSCs [54] (Additional file 7: Fig. S6) showed no differences between mutation carriers and isogenic corrected cells. Additionally, previous studies have shown that the neuronal proportion in ADAD brains seems to be lower compared to AD [21]. Together, this suggests that the *circPSEN1* association described in here might not have a neuronal origin. To date, there is no reference panel or single-cell circular RNA-seq data to test this hypothesis via digital deconvolution or differential expression analyses. Future studies using these and other tools can not only inform of the cell of origin but also whether the pathways are involved in vascular clearance, angiogenesis, or blood–brain barrier regulation, among others.

circ*PSEN1* might be a regulatory factor located at the top hierarchical levels of the dysregulation of the amyloid-beta pathway and leading to the neuroinflammatory status. Our results show that circ*PSEN1* is dysregulated in all ADAD cases, independent of the specific mutation. However, due to the limited sample size of *PSEN2* and *APP* carriers, we cannot disregard the possibility that this alteration is unique to *PSEN1* mutation carriers.

Additional analyses to understand the biological function of circ*PSEN1* and its relationship with *PSEN1* are needed, along with the study of circular and linear forms of *PSEN2* and *APP* in mutation carriers to elucidate the biological consequences of circular RNAs in ADAD in comparison to AD. If further replicated, circ*PSEN1* might be targeted to diminish neuroinflammation in ADAD individuals to delay the onset of the disease or slow down its progression.

This study includes the largest sample of ADAD brains analyzed to date. However, it is still a study with limited sample size, therefore limiting the statistical power of this analysis. Although our findings are novel and possibly biologically relevant, due to the limited knowledge about circular RNAs and their biological function, along with the relationship between linear and circular forms of the same gene, we cannot claim any causal involvement of circ*PSEN1* with ADAD or AD.

## Conclusions

In conclusion, our circ*PSEN1* differential expression analysis has shown significant differences in the expression of circ*PSEN1* that are unique to ADAD, and independent of gene mutation. Due to the biological function previously ascribed to circular RNAs and our *in-silico* analyses, we hypothesize that this finding might be related to neuroinflammatory events that lead or that are caused by the accumulation of amyloid-beta. Future studies aimed at understanding the biological function of circ*PSEN1* might lead to a better understanding of its pathological involvement with ADAD and its potential as drug-target.

## Supplementary Information

**Additional file 1.** Supplementary Table S1, S2, S3, S4, S5, S6.

**Additional file 2.** Supplementary Fig. S1.

**Additional file 3.** Supplementary Fig. S2.

**Additional file 4.** Supplementary Fig. S3.

**Additional file 5.** Supplementary Fig. S4.

**Additional file 6.** Supplementary Fig. S5.

**Additional file 7.** Supplementary Fig. S6.

## Data Availability

Discovery dataset data corresponding to the participants who enrolled in the Knight-ADRC can be downloaded at the NIAGADS Knight ADRC collection (NG00083). Additional Knight-ADRC participants and DIAN data is available upon request to qualified researchers. Finally, MSBB dataset is publicly available in Synapse (syn3159438).

## Abbreviations

ADAD: Autosomal-dominant Alzheimer's disease
AD: Alzheimer's disease
QC: Quality control
FC: Fold change
NFT: Neurofibrillary tangle
*APP*: Amyloid precursor protein
*PSEN1*: Presenilin 1
*PSEN2*: Presenilin 2
CDR: Clinical dementia rating
NHW: Non-hispanic white
DIAN: Dominantly inherited Alzheimer network
MSBB: Mount Sinai Brain Bank
BM: Brodmann area
circ*PSEN1*: Circular *PSEN1*
DE: Differential expression
PMI: Postmortem interval
TIN: Transcript integrity number
KEGG: Kyoto Encyclopedia of genes and genomes
ROC: Receiver operating characteristic curve
AUC: Areas under the ROC curve
JAK2: Janus kinase 2
TREM2: Triggering receptor expressed on myeloid 2

## References

[1] https://www.alz.org/media/documents/alzheimers-facts-and-figures-infographic.pdf

[2] LaFerla FM, Oddo S (2005). Alzheimer's disease: Abeta, tau and synaptic dysfunction. Trends Mol Med.

[3] Ibanez L, Cruchaga C, Fernandez MV (2021). Advances in genetic and molecular understanding of Alzheimer's disease. Genes.

[4] Lin YS, Cheng CY, Liao YC, Hong CJ, Fuh JL (2020). Mutational analysis in familial Alzheimer's disease of Han Chinese in Taiwan with a predominant mutation PSEN1 p.Met146Ile. Sci Rep.

[5] Bateman RJ, Aisen PS, De Strooper B, Fox NC, Lemere CA, Ringman JM, Salloway S, Sperling RA, Windisch M, Xiong C (2011). Autosomal-dominant Alzheimer's disease: a review and proposal for the prevention of Alzheimer's disease. Alzheimer's Res Ther.

[6] Bekris LM, Yu CE, Bird TD, Tsuang DW (2010). Genetics of Alzheimer disease. J Geriatr Psychiatry Neurol.

[7] Hsu S, Gordon BA, Hornbeck R, Norton JB, Levitch D, Louden A, Ziegemeier E, Laforce R, Chhatwal J, Day GS (2018). Discovery and validation of autosomal dominant Alzheimer's disease mutations. Alzheimer's Res Ther.

[8] Karch CM, Hernandez D, Wang JC, Marsh J, Hewitt AW, Hsu S, Norton J, Levitch D, Donahue T, Sigurdson W (2018). Human fibroblast and stem cell resource from the Dominantly Inherited Alzheimer Network. Alzheimer's Res Ther.

[9] Fernandez MV, Kim JH, Budde JP, Black K, Medvedeva A, Saef B, Deming Y, Del-Aguila J, Ibanez L, Dube U (2017). Analysis of neurodegenerative Mendelian genes in clinically diagnosed Alzheimer Disease. PLoS Genet.

[10] Ryan NS, Nicholas JM, Weston PSJ, Liang Y, Lashley T, Guerreiro R, Adamson G, Kenny J, Beck J, Chavez-Gutierrez L (2016). Clinical phenotype and genetic associations in autosomal dominant familial Alzheimer's disease: a case series. Lancet Neurol.

[11] Ringman JM, Monsell S, Ng DW, Zhou Y, Nguyen A, Coppola G, Van Berlo V, Mendez MF, Tung S, Weintraub S (2016). Neuropathology of autosomal dominant Alzheimer disease in the National Alzheimer Coordinating Center database. J Neuropathol Exp Neurol.

[12] Boon BDC, Bulk M, Jonker AJ, Morrema THJ, van den Berg E, Popovic M, Walter J, Kumar S, van der Lee SJ, Holstege H (2020). The coarse-grained plaque: a divergent Abeta plaque-type in early-onset Alzheimer's disease. Acta Neuropathol.

[13] Cignarella F, Filipello F, Bollman B, Cantoni C, Locca A, Mikesell R, Manis M, Ibrahim A, Deng L, Benitez BA (2020). TREM2 activation on microglia promotes myelin debris clearance and remyelination in a model of multiple sclerosis. Acta Neuropathol.

[14] Guerreiro R, Wojtas A, Bras J, Carrasquillo M, Rogaeva E, Majounie E, Cruchaga C, Sassi C, Kauwe JS, Younkin S (2013). TREM2 variants in Alzheimer's disease. N Engl J Med.

[15] Ewers M, Franzmeier N, Suarez-Calvet M, Morenas-Rodriguez E, Caballero MAA, Kleinberger G, Piccio L, Cruchaga C, Deming Y, Dichgans M (2019). Increased soluble TREM2 in cerebrospinal fluid is associated with reduced cognitive and clinical decline in Alzheimer's disease. Sci Transl Med.

[16] Deming Y, Filipello F, Cignarella F, Cantoni C, Hsu S, Mikesell R, Li Z, Del-Aguila JL, Dube U, Farias FG (2019). The MS4A gene cluster is a key modulator of soluble TREM2 and Alzheimer's disease risk. Sci Transl Med.

[17] Del-Aguila JL, Li Z, Dube U, Mihindukulasuriya KA, Budde JP, Fernandez MV, Ibanez L, Bradley J, Wang F, Bergmann K (2019). A single-nuclei RNA sequencing study of Mendelian and sporadic AD in the human brain. Alzheimer's Res Ther.

[18] Del-Aguila JL, Benitez BA, Li Z, Dube U, Mihindukulasuriya KA, Budde JP, Farias FHG, Fernandez MV, Ibanez L, Jiang S (2019). TREM2 brain transcript-specific studies in AD and TREM2 mutation carriers. Mol Neurodegener.

[19] Ringman JM, Goate A, Masters CL, Cairns NJ, Danek A, Graff-Radford N, Ghetti B, Morris JC (2014). Dominantly inherited Alzheimer N: genetic heterogeneity in Alzheimer disease and implications for treatment strategies. Curr Neurol Neurosci Rep.

[20] Dube U, Del-Aguila JL, Li Z, Budde JP, Jiang S, Hsu S, Ibanez L, Fernandez MV, Farias F, Norton J (2019). An atlas of cortical circular RNA expression in Alzheimer disease brains demonstrates clinical and pathological associations. Nat Neurosci.

[21] Li Z, Del-Aguila JL, Dube U, Budde J, Martinez R, Black K, Xiao Q, Cairns NJ, Dougherty JD, Dominantly Inherited Alzheimer Network (2018). Genetic variants associated with Alzheimer's disease confer different cerebral cortex cell-type population structure. Genome Med.

[22] LaFerla FM, Green KN (2012). Animal models of Alzheimer disease. Cold Spring Harb Perspect Med.

[23] Barrett SP, Salzman J (2016). Circular RNAs: analysis, expression and potential functions. Development.

[24] Li X, Yang L, Chen LL (2018). The biogenesis, functions, and challenges of circular RNAs. Mol Cell.

[25] Mehta SL, Dempsey RJ, Vemuganti R (2020). Role of circular RNAs in brain development and CNS diseases. Prog Neurobiol.

[26] Ravanidis S, Bougea A, Karampatsi D, Papagiannakis N, Maniati M, Stefanis L, Doxakis E (2021). Differentially expressed circular RNAs in peripheral blood mononuclear cells of patients with Parkinson's disease. Mov Disord Off J Mov Disord Soc.

[27] Hanan M, Simchovitz A, Yayon N, Vaknine S, Cohen-Fultheim R, Karmon M, Madrer N, Rohrlich TM, Maman M, Bennett ER (2020). A Parkinson's disease CircRNAs resource reveals a link between circSLC8A1 and oxidative stress. EMBO Mol Med.

[28] Morris JC (1993). The clinical dementia rating (CDR): current version and scoring rules. Neurology.

[29] Dobin A, Davis CA, Schlesinger F, Drenkow J, Zaleski C, Jha S, Batut P, Chaisson M, Gingeras TR (2013). STAR: ultrafast universal RNA-seq aligner. Bioinformatics.

[30] http://broadinstitute.github.io/picard/

[31] Patro R, Duggal G, Love MI, Irizarry RA, Kingsford C (2017). Salmon provides fast and bias-aware quantification of transcript expression. Nat Methods.

[32] Cheng J, Metge F, Dieterich C (2016). Specific identification and quantification of circular RNAs from sequencing data. Bioinformatics.

[33] Love MI, Huber W, Anders S (2014). Moderated estimation of fold change and dispersion for RNA-seq data with DESeq2. Genome Biol.

[34] Wang L, Nie J, Sicotte H, Li Y, Eckel-Passow JE, Dasari S, Vedell PT, Barman P, Wang L, Weinshiboum R (2016). Measure transcript integrity using RNA-seq data. BMC Bioinform.

[35] Dudekula DB, Panda AC, Grammatikakis I, De S, Abdelmohsen K, Gorospe M (2016). CircInteractome: a web tool for exploring circular RNAs and their interacting proteins and microRNAs. RNA Biol.

[36] Vlachos IS, Zagganas K, Paraskevopoulou MD, Georgakilas G, Karagkouni D, Vergoulis T, Dalamagas T, Hatzigeorgiou AG (2015). DIANA-miRPath v3.0: deciphering microRNA function with experimental support. Nucleic Acids Res.

[37] You X, Vlatkovic I, Babic A, Will T, Epstein I, Tushev G, Akbalik G, Wang M, Glock C, Quedenau C (2015). Neural circular RNAs are derived from synaptic genes and regulated by development and plasticity. Nat Neurosci.

[38] Grimson A, Farh KK, Johnston WK, Garrett-Engele P, Lim LP, Bartel DP (2007). MicroRNA targeting specificity in mammals: determinants beyond seed pairing. Mol Cell.

[39] Ashwal-Fluss R, Meyer M, Pamudurti NR, Ivanov A, Bartok O, Hanan M, Evantal N, Memczak S, Rajewsky N, Kadener S (2014). circRNA biogenesis competes with pre-mRNA splicing. Mol Cell.

[40] Hsieh YC, Guo C, Yalamanchili HK, Abreha M, Al-Ouran R, Li Y, Dammer EB, Lah JJ, Levey AI, Bennett DA (2019). Tau-mediated disruption of the spliceosome triggers cryptic RNA splicing and neurodegeneration in Alzheimer's disease. Cell Rep.

[41] Nuzzo D, Inguglia L, Walters J, Picone P, Di Carlo M (2017). A shotgun proteomics approach reveals a new toxic role for Alzheimer's disease Abeta peptide: spliceosome impairment. J Proteome Res.

[42] Inestrosa NC, Montecinos-Oliva C, Fuenzalida M (2012). Wnt signaling: role in Alzheimer disease and schizophrenia. J Neuroimmune Pharmacol Off J Soc NeuroImmune Pharmacol.

[43] Zhang L, Qi Z, Li J, Li M, Du X, Wang S, Zhou G, Xu B, Liu W, Xi S (2021). Roles and mechanisms of Axon-guidance molecules in Alzheimer's disease. Mol Neurobiol.

[44] Wang SP, Wang LH (2016). Disease implication of hyper-Hippo signalling. Open Biol.

[45] Yang Y, Zhang Z (2020). Microglia and Wnt pathways: prospects for inflammation in Alzheimer's disease. Front Aging Neurosci.

[46] Keller A, Leidinger P, Vogel B, Backes C, ElSharawy A, Galata V, Mueller SC, Marquart S, Schrauder MG, Strick R (2014). miRNAs can be generally associated with human pathologies as exemplified for miR-144. BMC Med.

[47] Li K, Zhang J, Ji C, Wang L (2016). MiR-144-3p and its target gene beta-amyloid precursor protein regulate 1-methyl-4-phenyl-1,2–3,6-tetrahydropyridine-induced mitochondrial dysfunction. Mol Cells.

[48] Wang R, Zhang J (2020). Clinical significance of miR-433 in the diagnosis of Alzheimer's disease and its effect on Abeta-induced neurotoxicity by regulating JAK2. Exp Gerontol.

[49] Cha DJ, Mengel D, Mustapic M, Liu W, Selkoe DJ, Kapogiannis D, Galasko D, Rissman RA, Bennett DA, Walsh DM (2019). miR-212 and miR-132 are downregulated in neurally derived plasma exosomes of Alzheimer's patients. Front Neurosci.

[50] Dangla-Valls A, Molinuevo JL, Altirriba J, Sanchez-Valle R, Alcolea D, Fortea J, Rami L, Balasa M, Munoz-Garcia C, Ezquerra M (2017). CSF microRNA profiling in Alzheimer's disease: a screening and validation study. Mol Neurobiol.

[51] Liu S, Li XM, Yuan JB, Li LL, Wang C, Lin XM, Miao X, Shi ZC (2021). MiR-665 inhibits inflammatory response in microglia following spinal cord injury by targeting TREM2. Eur Rev Med Pharmacol Sci.

[52] Heneka MT, Carson MJ, El Khoury J, Landreth GE, Brosseron F, Feinstein DL, Jacobs AH, Wyss-Coray T, Vitorica J, Ransohoff RM (2015). Neuroinflammation in Alzheimer's disease. Lancet Neurol.

[53] Lee WS, Lee WH, Bae YC, Suk K (2019). Axon guidance molecules guiding neuroinflammation. Exp Neurobiol.

[54] Kwart D, Gregg A, Scheckel C, Murphy EA, Paquet D, Duffield M, Fak J, Olsen O, Darnell RB, Tessier-Lavigne M (2019). A large panel of isogenic APP and PSEN1 Mutant Human iPSC neurons reveals shared endosomal abnormalities mediated by APP beta-CTFs, not Abeta. Neuron.

