# Supplementary material for: Circular RNA detection identifies circPSEN1 alterations in brain specific to autosomal dominant Alzheimer's disease
Source: Acta Neuropathol Commun. 2022 Mar 4;10:29. doi: 10.1186/s40478-022-01328-5 (PMC8895634; doi:10.1186/s40478-022-01328-5)
Supplement: Supplementary file 1 — Additional file 1. Supplementary Table S1, S2, S3, S4, S5, S6. [file 40478_2022_1328_MOESM1_ESM.docx]

**Supplementary Table 1.** Neuropathological characteristics of the discovery and replication datasets

|  |  | **Discovery** | | | **Replication** | | |
| --- | --- | --- | --- | --- | --- | --- | --- |
|  |  | **ADAD** | **AD** | **Controls** | **ADAD** | **AD** | **Controls** |
| **Sample Size** |  | 17 | 59 | 10 | 4 | 194 | 13 |
| **NIA-Reagan Criteria** | High  Intermediate  Low  Criteria not met  Not Done  Missing/unknown | 16  0  0  0  0  1 | 32  4  3  0  14  6 | 0  0  0  8  2  0 | 1  0  0  0  2  1 | 79  16  10  1  79  9 | 0  1  2  5  2  3 |
| **Braak NFT** | Stage I  Stage II  Stage III  Stage IV  Stage V  Stage VI  Degeneration not present  Not assessed  Missing/unknown | 0  0  0  0  1  15  0  0  1 | 1  2  0  4  18  19  0  14  1 | 4  3  0  1  0  0  0  2  0 | 0  0  0  0  0  2  0  2  0 | 0  5  7  7  31  57  0  79  8 | 1  5  5  0  0  0  0  2  0 |
| **Neuritic Plaque Density** | Frequent  Moderate  Sparse  No neuritic plaques  Not assessed  Missing/unknown | 16  0  0  0  0  1 | 30  7  2  0  14  6 | 0  0  0  8  2  0 | 1  0  0  0  2  1 | 74  20  10  1  80  9 | 0  1  1  6  2  3 |
| **McKeith classification of Lewy body disease** | Brainstem Predominant  Intermediate type  Diffuse (neocortical) type  Unspecified  No Lewy Bodies  Not assessed  Missing/Unknown | 0  1  4  3  7  0  2 | 0  1  11  6  35  0  6 | 0  0  0  0  10  0  0 | 0  0  0  0  3  0  1 | 0  18  14  24  129  0  9 | 1  0  1  1  7  0  3 |

ADAD= ADAD=Autosomal Dominant Alzheimer's Disease; AD=Sporadic Alzheimer's Disease; NFT=NeuroFilament.

**Supplementary Table 2.** Demographic characteristics of the Mount Sinai Brain Bank (MSBB) in the different brain regions

|  | **BM10** | | **BM22** | | **BM36** | | **BM44** | |
| --- | --- | --- | --- | --- | --- | --- | --- | --- |
|  | **AD** | **Controls** | **AD** | **Controls** | **AD** | **Controls** | **AD** | **Controls** |
| **N** | 143 | 29 | 134 | 26 | 123 | 24 | 132 | 25 |
| **Sex**  **(% males)** | 32% | 31% | 0.37 | 0.31 | 0.33 | 0.33 | 0.33 | 0.36 |
| **Age at Onset**  **(years)** | 87.0  (71.1-90.0) | - | 85.0  (71.7-90.0) | - | 87.0  (71.1-90.0) | - | 87.0  (72.0-90.0) | - |
| **PMI**  **(hours)** | 5.0  (2.0-15.0) | 10.0  (4.0-19.6) | 5.0  (2.0-15.3) | 8.5  (4.3-19.8) | 5.0  (2.0-15.0) | 8.0  (3.2-19.0) | 5.0  (2.0-15.0) | 9.0  (4.0-19.8) |

BM= Brodmann; AD=Sporadic Alzheimer's Disease; PMI=Post-Mortem Interval; PMI and Age at Onset is expressed as median age (95% Inter Quartile Interval).

**Supplementary Table 3.** Differential expression of aggregate circ*PSEN1* and the species identified in the four MSBB brain regions

|  | **Aggregate circ*PSEN1*** | | **hsa_circ_0008521** | | **hsa_circ_0003848** | |
| --- | --- | --- | --- | --- | --- | --- |
|  | **log_2_FoldChange** | **P value** | **log_2_FoldChange** | **P value** | **log_2_FoldChange** | **P value** |
| **BM10** | 0.029 | 0.877 | - | - | -0.292 | 0.397 |
| **BM22** | 0.029 | 0.901 | 0.448 | 0.374 | 0.317 | 0.381 |
| **BM36** | 0.019 | 0.898 | 0.193 | 0.363 | -0.063 | 0.743 |
| **BM44** | 0.215 | 0.143 | 0.434 | 0.061 | 0.548 | 0.194 |

BM= Brodmann Area; *PSEN1*=Presinilin1

**Supplementary Table 4.** Differential expression of circ*PSEN1* when adding, Braak NFT, or age at death to the model to test for the independency of linear and circular forms of *PSEN1*

| **Model** | **Sample Size** | | **circ*PSEN1* DE** | |
| --- | --- | --- | --- | --- |
|  | **ADAD** | **AD** | **log_2_FoldChange** | **P value** |
| Status+Sex+PMI+TIN+Dataset+BraakNFT | 15 | 143 | 0.045 | 0.486 |
| Status+Sex+PMI+TIN+Dataset+Age at Death | 21 | 253 | -0.173 | 0.126 |

DE=Differential Expression, ADAD=Autosomal Dominant Alzheimer's Disease; AD=Sporadic Alzheimer's Disease; PMI=Post-Mortem Interval; TIN=Transcript Integrity Number; NFT=NeuroFiblirary Tangles

**Supplementary Table 5.** KEGG pathways identified by DIANA mirPath software to be significantly associated with the miRNA identified by the Circular RNA Interactome to bind circ*PSEN1*.

| **KEGG pathway** | **P value** | **KEGG pathway** | **P value** |
| --- | --- | --- | --- |
| Proteoglycans in cancer | 6.81E-09 | Adrenergic signaling in cardiomyocytes | 4.47E-03 |
| Axon guidance | 3.39E-07 | Thyroid hormone signaling pathway | 4.47E-03 |
| Hippo signaling pathway | 7.38E-07 | Endometrial cancer | 4.47E-03 |
| Signaling pathways regulating pluripotency of stem cells | 8.99E-06 | Transcriptional misregulation in cancer | 4.47E-03 |
| Lysine degradation | 2.48E-05 | Focal adhesion | 5.04E-03 |
| Pathways in cancer | 1.95E-04 | Neurotrophin signaling pathway | 5.26E-03 |
| ErbB signaling pathway | 3.25E-04 | Non-small cell lung cancer | 6.95E-03 |
| Wnt signaling pathway | 5.58E-04 | Oxytocin signaling pathway | 7.00E-03 |
| Dorso-ventral axis formation | 8.02E-04 | AMPK signaling pathway | 7.98E-03 |
| Hepatitis B | 8.09E-04 | GABAergic synapse | 7.98E-03 |
| Prostate cancer | 8.40E-04 | Glutamatergic synapse | 7.98E-03 |
| Glioma | 8.40E-04 | Adherens junction | 9.07E-03 |
| TGF-beta signaling pathway | 9.47E-04 | Nicotine addiction | 0.01 |
| MAPK signaling pathway | 9.55E-04 | Dopaminergic synapse | 0.01 |
| PI3K-Akt signaling pathway | 1.98E-03 | cAMP signaling pathway | 0.02 |
| Colorectal cancer | 3.16E-03 | Long-term potentiation | 0.02 |
| Ras signaling pathway | 3.16E-03 | Renal cell carcinoma | 0.02 |
| FoxO signaling pathway | 3.18E-03 | Regulation of actin cytoskeleton | 0.02 |
| Biotin metabolism | 4.47E-03 | Circadian entrainment | 0.03 |
| Pancreatic cancer | 4.47E-03 | Melanoma | 0.03 |
| Chronic myeloid leukemia | 4.47E-03 | Rap1 signaling pathway | 0.03 |
| mTOR signaling pathway | 4.47E-03 | Retrograde endocannabinoid signaling | 0.04 |
| Amphetamine addiction | 4.47E-03 |  |  |

**Supplementary Table 6.** Common genes within the KEGG pathways identified by DIANA mirPath software and their differential expression in brains of ADAD, AD and, controls for the discovery and the replication datasets

|  | Discovery | | | | | | | | Replication | | | | | | | |
| --- | --- | --- | --- | --- | --- | --- | --- | --- | --- | --- | --- | --- | --- | --- | --- | --- |
|  | **ADADvsCO** | | | **ADADvsAD** | | | **ADvsCO** | | **ADADvsCO** | | | **ADADvsAD** | | | **ADvsCO** | |
| Genes | **log_2_FC** | **P value** | **log_2_FC** | | **P value** | **log_2_FC** | | **P value** | **log_2_FC** | **P value** | **log_2_FC** | | **P value** | **log_2_FC** | | **P value** |
| AKT2 | -0.0724 | 6.39E-01 | 0.0739 | | 4.49E-01 | -0.1434 | | 2.37E-01 | -0.1171 | 6.34E-01 | -0.2327 | | 1.55E-01 | 0.1320 | | 1.37E-01 |
| GRB2 | 0.0107 | 8.95E-01 | 0.0010 | | 9.84E-01 | -0.0087 | | 8.99E-01 | 0.0394 | 7.29E-01 | 0.1267 | | 2.22E-01 | -0.0709 | | 1.93E-01 |
| WNT9B | 0.1527 | 4.46E-01 | 0.0137 | | 9.21E-01 | 0.1663 | | 3.55E-01 | 0.5069 | 4.16E-01 | 0.8533 | | 3.92E-02 | -0.3010 | | 1.53E-01 |
| PRKCB | 0.2241 | 2.03E-01 | 0.1816 | | 2.38E-01 | -0.0038 | | 9.84E-01 | -0.2684 | 6.70E-01 | 0.3839 | | 2.53E-01 | -0.6003 | | 1.50E-03 |
| SMAD3 | -0.3122 | 1.96E-02 | -0.1087 | | 1.97E-01 | -0.1160 | | 2.78E-01 | 0.0578 | 8.47E-01 | -0.1531 | | 3.73E-01 | 0.0037 | | 9.70E-01 |
| WNT10B | 0.4645 | 1.89E-02 | 0.5156 | | 9.97E-04 | 0.0411 | | 8.37E-01 | 0.1361 | 8.74E-01 | 0.4250 | | 2.74E-01 | -0.3501 | | 1.14E-01 |
| KRAS | 0.2401 | 5.24E-02 | 0.1162 | | 2.21E-01 | 0.1113 | | 3.93E-01 | -0.0538 | 8.36E-01 | 0.2019 | | 3.13E-01 | -0.1617 | | 1.38E-01 |
| MAP2K1 | -0.0121 | 9.45E-01 | -0.0070 | | 9.55E-01 | -0.1054 | | 4.94E-01 | -0.2347 | 5.05E-01 | 0.2921 | | 2.91E-01 | -0.2701 | | 7.78E-02 |
| FZD4 | -0.2342 | 2.60E-01 | -0.2418 | | 3.74E-02 | 0.0248 | | 8.35E-01 | -0.5838 | 4.64E-02 | -0.6329 | | 1.35E-03 | -0.0140 | | 8.95E-01 |
| PIK3R5 | -1.0713 | 1.01E-04 | -0.5582 | | 1.15E-02 | -0.6184 | | 2.39E-02 | -0.4223 | 2.62E-01 | -0.6961 | | 5.72E-02 | 0.0117 | | 9.52E-01 |
| MAPK1 | -0.1731 | 3.46E-02 | -0.1258 | | 4.98E-02 | -0.0361 | | 6.64E-01 | 0.1213 | 6.07E-01 | 0.1652 | | 1.98E-01 | -0.0570 | | 4.27E-01 |
| WNT6 | -0.7467 | 2.25E-01 | -0.6210 | | 1.37E-01 | -0.6385 | | 2.73E-01 | -0.0388 | 9.61E-01 | -1.2207 | | 1.22E-01 | 1.1848 | | 5.60E-03 |
| CTNNB1 | -0.1878 | 2.36E-03 | -0.0837 | | 1.49E-01 | -0.0865 | | 2.43E-01 | 0.2385 | 1.91E-01 | 0.0818 | | 3.74E-01 | 0.1692 | | 7.80E-04 |
| FZD5 | -0.8505 | 5.23E-06 | -0.2045 | | 2.10E-01 | -0.6462 | | 2.37E-03 | -0.1610 | 7.17E-01 | -0.4072 | | 2.37E-01 | 0.3236 | | 7.57E-02 |
| WNT5A | -0.1663 | 3.67E-01 | 0.0032 | | 9.78E-01 | -0.1675 | | 2.02E-01 | 0.3245 | 3.59E-01 | -0.0031 | | 9.89E-01 | 0.3022 | | 7.92E-03 |
| SMAD4 | -0.6016 | 7.62E-07 | -0.2880 | | 1.17E-03 | -0.2960 | | 7.09E-03 | -0.1224 | 4.42E-01 | -0.1173 | | 3.66E-01 | -0.0067 | | 9.23E-01 |
| WNT2B | -0.0399 | 7.89E-01 | 0.0313 | | 7.18E-01 | 0.0198 | | 8.65E-01 | -0.2666 | 2.93E-01 | 0.0594 | | 7.71E-01 | -0.2839 | | 8.49E-03 |
| PIK3CG | -0.1939 | 4.56E-01 | 0.0374 | | 8.37E-01 | 0.0750 | | 7.34E-01 | -0.2287 | 3.92E-01 | -0.2002 | | 5.37E-01 | -0.0793 | | 6.39E-01 |
| WNT16 | 0.8150 | 5.74E-02 | 0.3080 | | 3.71E-01 | 0.2511 | | 5.15E-01 | -0.0566 | 9.44E-01 | 0.0844 | | 8.90E-01 | -0.4285 | | 1.82E-01 |
| PIK3CB | 0.2037 | 1.73E-01 | 0.1259 | | 2.29E-01 | 0.0468 | | 7.21E-01 | -0.1825 | 3.45E-01 | 0.1385 | | 4.86E-01 | -0.2253 | | 3.27E-02 |
| PRKCA | -0.1297 | 2.74E-01 | -0.2100 | | 1.19E-03 | 0.1436 | | 3.46E-02 | -0.0207 | 9.37E-01 | -0.0426 | | 7.61E-01 | -0.1032 | | 1.92E-01 |
| WNT7A | -0.1159 | 6.37E-01 | -0.2102 | | 1.87E-01 | -0.0324 | | 8.77E-01 | -1.1896 | 1.74E-02 | -0.2755 | | 3.60E-01 | -0.9619 | | 2.37E-08 |
| GSK3B | 0.1766 | 4.39E-02 | 0.1006 | | 2.28E-01 | 0.0691 | | 5.16E-01 | -0.0495 | 7.75E-01 | 0.2256 | | 1.69E-01 | -0.1830 | | 3.72E-02 |
| AKT3 | 0.2262 | 2.32E-02 | 0.0977 | | 2.36E-01 | 0.1178 | | 2.91E-01 | 0.0368 | 7.22E-01 | 0.2038 | | 1.41E-01 | -0.0873 | | 2.32E-01 |
| PIK3CA | 0.0970 | 3.85E-01 | 0.0426 | | 5.74E-01 | 0.0530 | | 6.02E-01 | -0.1083 | 4.30E-01 | 0.1183 | | 3.53E-01 | -0.1586 | | 1.88E-02 |
| RAF1 | -0.3583 | 1.22E-05 | -0.1794 | | 5.55E-04 | -0.2195 | | 2.22E-04 | -0.1442 | 2.62E-01 | -0.2037 | | 3.87E-02 | 0.0670 | | 1.96E-01 |
| WNT9A | -0.1131 | 8.09E-01 | 0.0392 | | 8.88E-01 | 0.0057 | | 9.86E-01 | -0.2204 | 7.69E-01 | -0.6735 | | 1.61E-01 | 0.1772 | | 4.97E-01 |
| FZD8 | -0.5680 | 3.83E-02 | -0.3553 | | 7.55E-02 | -0.1821 | | 4.65E-01 | -0.7128 | 1.31E-01 | -0.5984 | | 9.57E-02 | -0.3548 | | 6.93E-02 |
| FZD1 | -0.5506 | 1.36E-02 | -0.2030 | | 1.72E-01 | -0.3550 | | 4.23E-02 | -0.3247 | 4.83E-01 | -0.5988 | | 2.16E-02 | 0.1954 | | 1.77E-01 |
| FZD3 | 0.1273 | 3.53E-01 | 0.0992 | | 3.57E-01 | 0.0435 | | 7.50E-01 | -0.0343 | 9.25E-01 | 0.1636 | | 4.44E-01 | -0.1864 | | 1.10E-01 |
| FZD6 | 0.1862 | 3.27E-01 | 0.1782 | | 1.69E-01 | 0.0854 | | 6.02E-01 | 0.0788 | 8.32E-01 | -0.3292 | | 1.94E-01 | 0.2061 | | 1.22E-01 |
