# Supplementary material for: Circular RNA detection identifies circPSEN1 alterations in brain specific to autosomal dominant Alzheimer's disease
Source: Acta Neuropathol Commun. 2022 Mar 4;10:29. doi: 10.1186/s40478-022-01328-5 (PMC8895634; doi:10.1186/s40478-022-01328-5)

Supplementary Figure 1. Circular and linear *PSEN1* normalized counts in the Mount Sinai Brain Bank dataset. Panels A to D represent circ*PSEN1* and panels E to H linear *PSEN1* (BM10, BM22, BM36 and BM44 respectively).

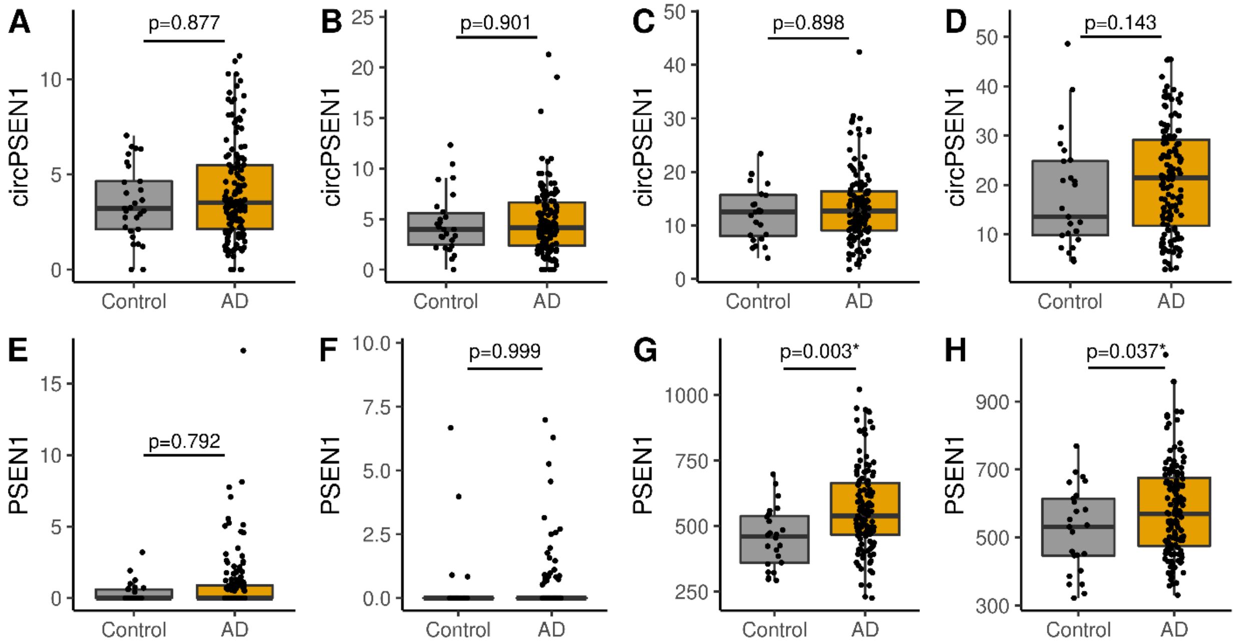

Supplement: Supplementary file 2 — Additional file 2. Supplementary Fig. S1. [file 40478_2022_1328_MOESM2_ESM.pdf]
