# Supplementary material for: Circular RNA detection identifies circPSEN1 alterations in brain specific to autosomal dominant Alzheimer's disease
Source: Acta Neuropathol Commun. 2022 Mar 4;10:29. doi: 10.1186/s40478-022-01328-5 (PMC8895634; doi:10.1186/s40478-022-01328-5)

**Supplementary Figure 2.** Comparison of the circular *PSEN1* normalized counts for the three main *circPSEN1* species S1 - hsa_circ_0008521 (Panel A in discovery, Panel B in replication), S2-hsa_circ_0003848 (Panel C in discovery, Panel D in replication), and S5 - hsa_circ_0002564 (Panel E in discovery, Panel F in replication) for Controls (grey), AD (Alzheimer's disease - ocher) and ADAD (autosomal dominant Alzheimer's disease - blue)

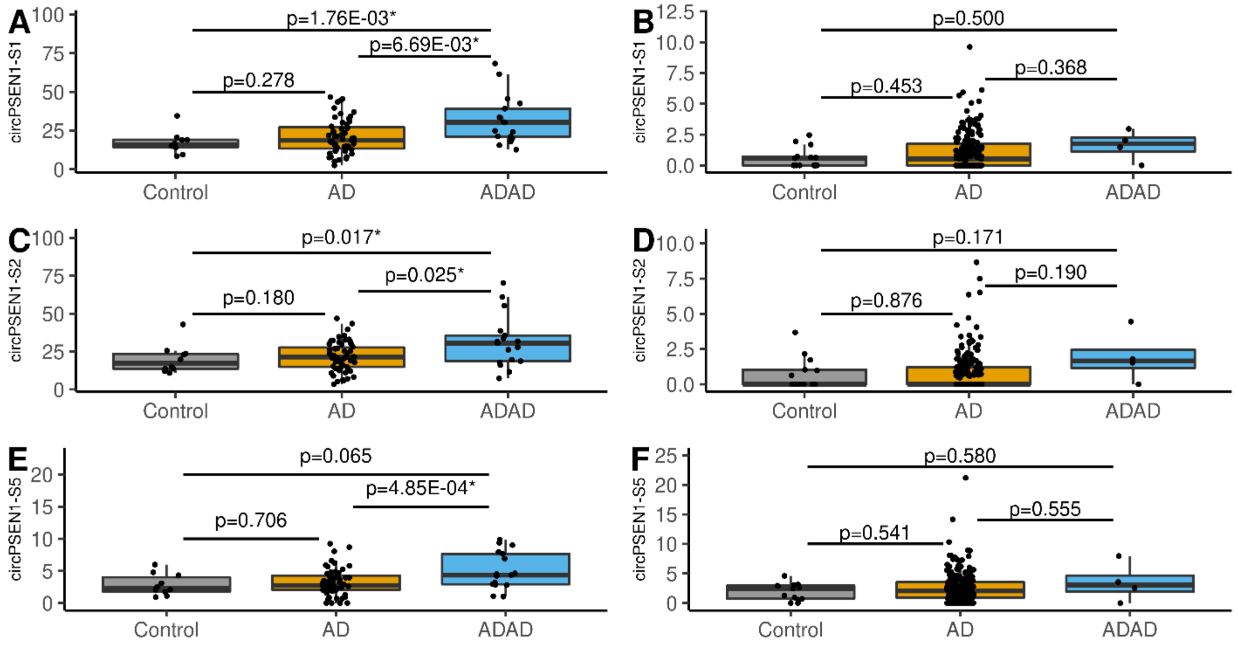

Supplement: Supplementary file 3 — Additional file 3. Supplementary Fig. S2. [file 40478_2022_1328_MOESM3_ESM.pdf]
