# Supplementary material for: Circular RNA detection identifies circPSEN1 alterations in brain specific to autosomal dominant Alzheimer's disease
Source: Acta Neuropathol Commun. 2022 Mar 4;10:29. doi: 10.1186/s40478-022-01328-5 (PMC8895634; doi:10.1186/s40478-022-01328-5)

Supplementary Figure 3. Correlation of circular *PSEN1* normalized counts and linear *PSEN1* normalized counts in ADAD (blue), AD (yellow), and controls (gray) for the discovery (Panel A) and replication (Panel B) Datasets

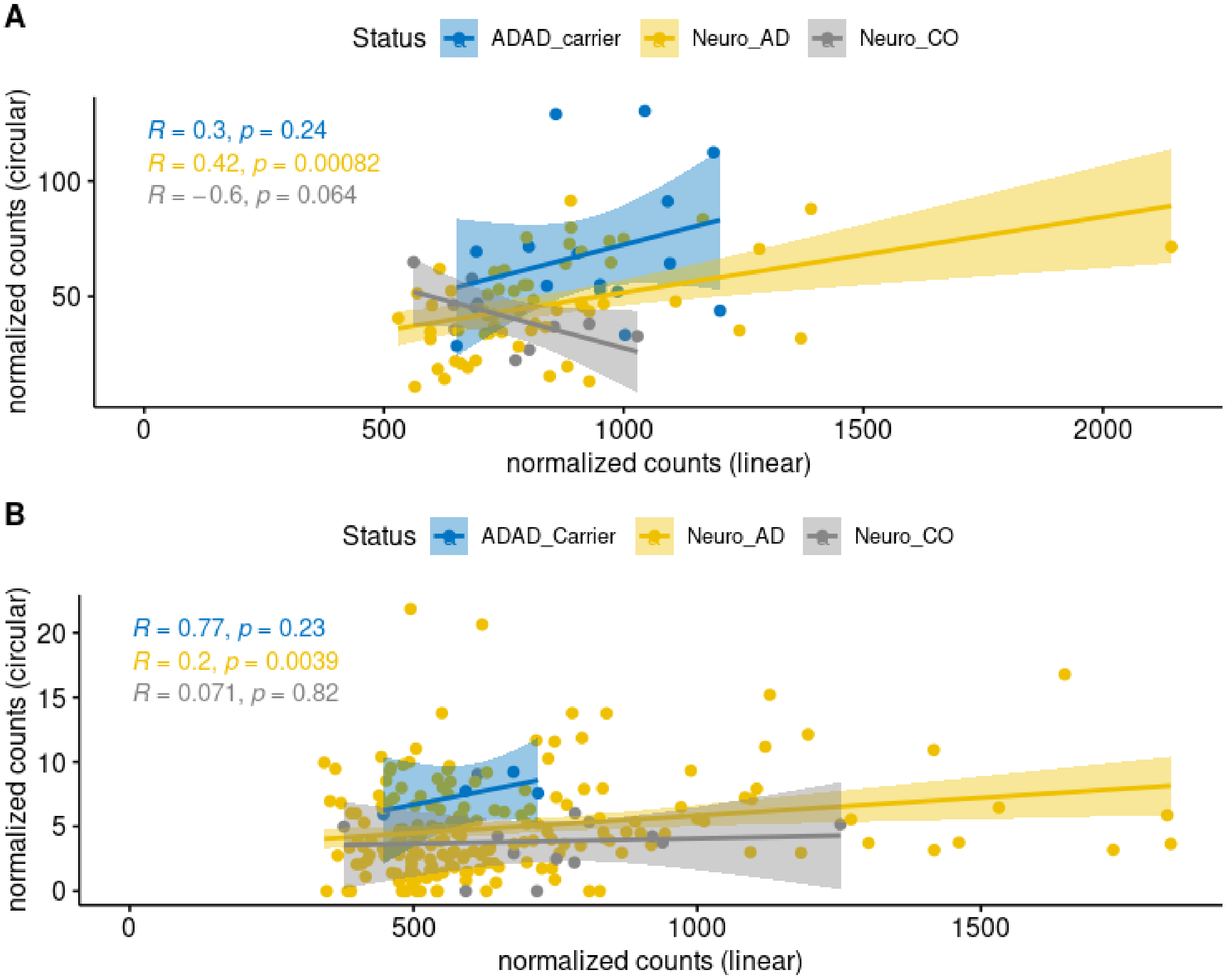

Supplement: Supplementary file 4 — Additional file 4. Supplementary Fig. S3. [file 40478_2022_1328_MOESM4_ESM.pdf]
