# Supplementary material for: Circular RNA detection identifies circPSEN1 alterations in brain specific to autosomal dominant Alzheimer's disease
Source: Acta Neuropathol Commun. 2022 Mar 4;10:29. doi: 10.1186/s40478-022-01328-5 (PMC8895634; doi:10.1186/s40478-022-01328-5)

Supplementary Figure 4. Correlation of circular *PSEN1* normalized counts of the three main species and linear *PSEN1* normalized counts in ADAD (blue), AD (yellow), and controls (gray). S1 - hsa_circ_0008521 in the discovery (Panel A) and the replication (Panel B), S2 - hsa_circ_0003848 in the discovery (Panel C) and the replication (Panel D), S5 - hsa_circ_0002564 in the discovery (Panel E) and the replication (Panel F).

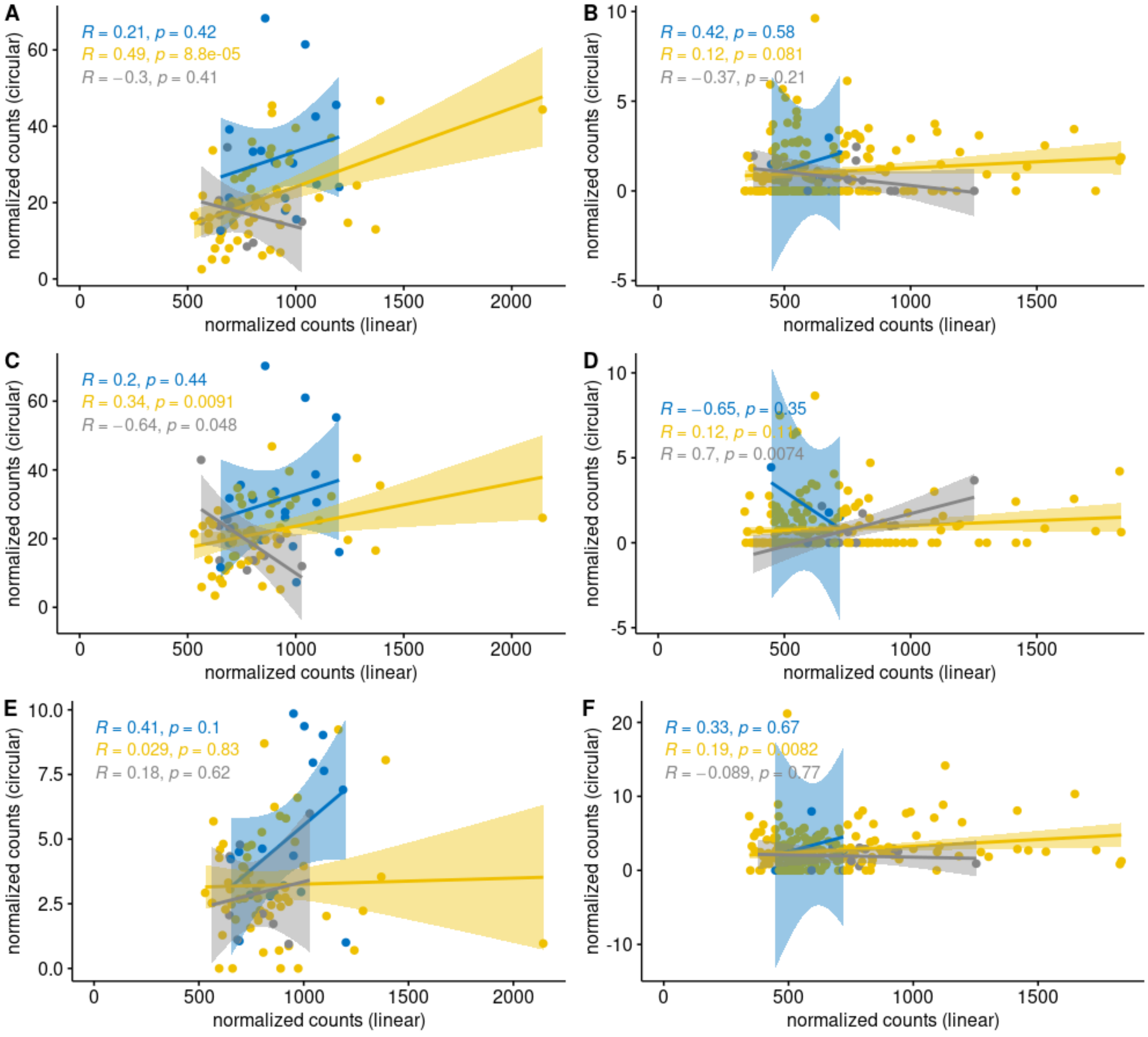

Supplement: Supplementary file 5 — Additional file 5. Supplementary Fig. S4. [file 40478_2022_1328_MOESM5_ESM.pdf]
