# Supplementary material for: Circular RNA detection identifies circPSEN1 alterations in brain specific to autosomal dominant Alzheimer's disease
Source: Acta Neuropathol Commun. 2022 Mar 4;10:29. doi: 10.1186/s40478-022-01328-5 (PMC8895634; doi:10.1186/s40478-022-01328-5)

Supplementary Figure 5. ROCs corresponding to the binomial regression models using different circ*PSEN1* counts (aggregate - red, hsa_circ_0008521 - blue, hsa_circ_0003848 - green) for classifying AD vs Controls (Panel A in discovery and Panel B in replication), ADAD vs AD (Panel C in discovery and Panel D in replication), ADAD vs Controls (Panel E in discovery and Panel F in replication).

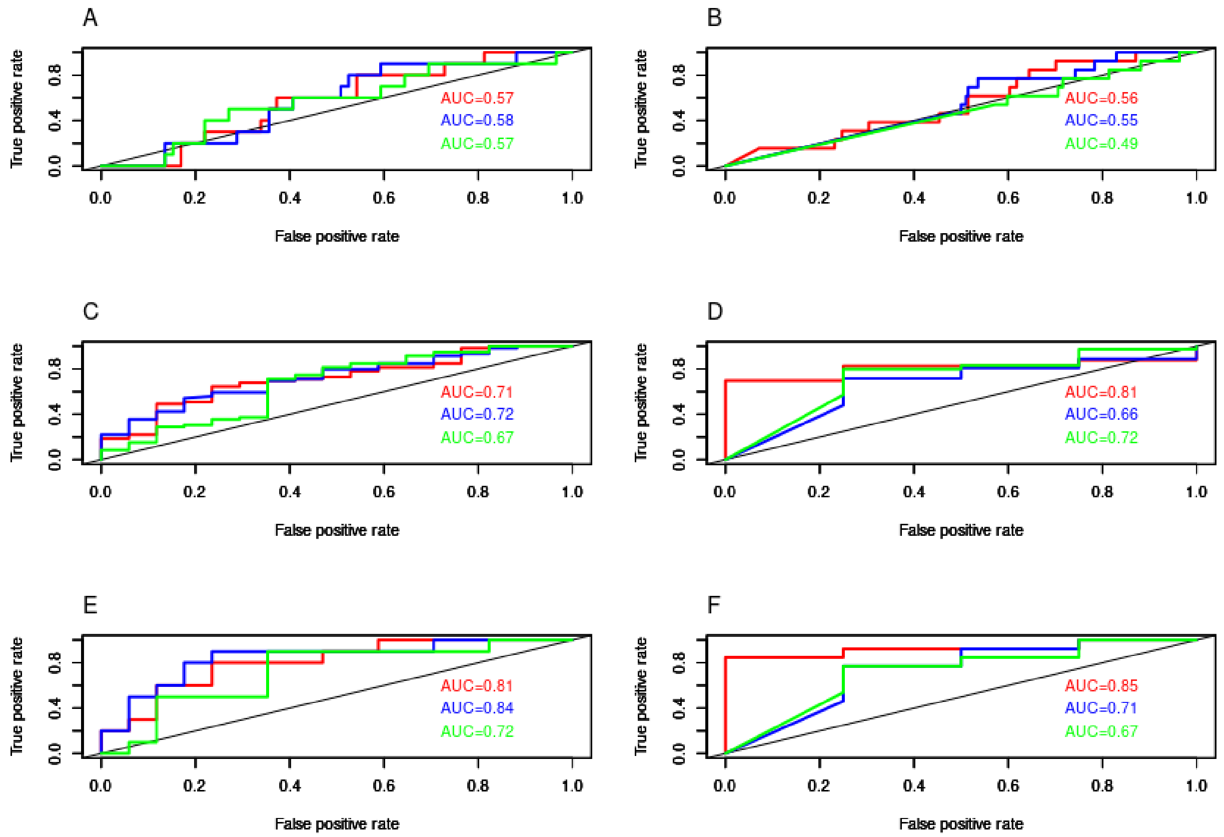

Supplement: Supplementary file 6 — Additional file 6. Supplementary Fig. S5. [file 40478_2022_1328_MOESM6_ESM.pdf]
