# Supplementary material for: Circular RNA detection identifies circPSEN1 alterations in brain specific to autosomal dominant Alzheimer's disease
Source: Acta Neuropathol Commun. 2022 Mar 4;10:29. doi: 10.1186/s40478-022-01328-5 (PMC8895634; doi:10.1186/s40478-022-01328-5)

Supplementary Figure 6. Comparison of the circular *PSEN1* normalized counts in the IPSC dataset [54] for isogenic controls (grey), mutation carriers (ocher), no significant difference between the two groups was observed (p=0.730).

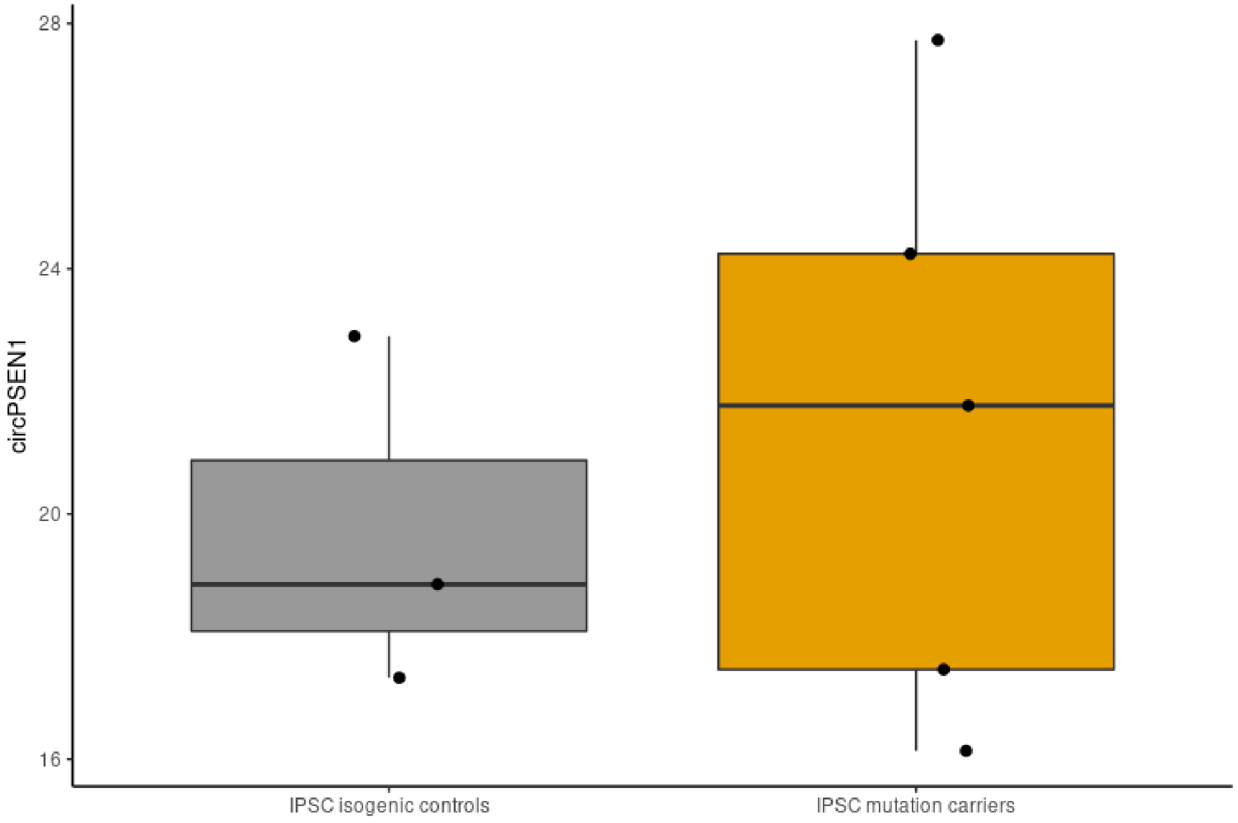

Supplement: Supplementary file 7 — Additional file 7. Supplementary Fig. S6. [file 40478_2022_1328_MOESM7_ESM.pdf]
